# Noradrenalin effectively rescues mice from blast lung injury caused by laser-induced shock waves

**DOI:** 10.1186/s40635-015-0069-7

**Published:** 2015-12-10

**Authors:** Hiroki Miyawaki, Daizoh Saitoh, Kohsuke Hagisawa, Midori Noguchi, Shunichi Sato, Manabu Kinoshita, Hiromi Miyazaki, Yasushi Satoh, Nahoko Harada, Toshihisa Sakamoto

**Affiliations:** Department of Traumatology and Critical Care Medicine, National Defense Medical College Hospital, 3-2 Namiki, Tokorozawa, 359-8513 Japan; Division of Traumatology, Research Institute, National Defense Medical College, 3-2 Namiki, Tokorozawa, 359-8513 Japan; Division of Physiology, National Defense Medical College, 3-2 Namiki, Tokorozawa, 359-8513 Japan; Division of Biomedical Information Sciences, National Defense Medical College, 3-2 Namiki, Tokorozawa, 359-8513 Japan; Department of Immunology and Microbiology, National Defense Medical College, 3-2 Namiki, Tokorozawa, 359-8513 Japan; Department of Anesthesiology, National Defense Medical College, 3-2 Namiki, Tokorozawa, 359-8513 Japan; Division of Nursing, School of Medicine, National Defense Medical College, 3-2 Namiki, Tokorozawa, 359-8513 Japan

**Keywords:** Blast lung injury, Initial phase, Laser-induced shock wave, Noradrenalin, Peripheral vasoconstriction

## Abstract

**Background:**

Blast lung injuries (BLI) caused by blast waves are extremely critical in the prehospital setting, and hypotension is thought to be the main cause of death in such cases. The present study aimed to elucidate the pathophysiology of severe BLI using laser-induced shock wave (LISW) and identify the initial treatment.

**Methods:**

The current investigation comprised two parts. For the validation study, mice were randomly allocated to groups that received a single shot of 1.2, 1.3, or 1.4 J/cm^2^ LISW to both lungs. The survival rates, systolic blood pressure (sBP), heart rate (HR), peripheral oxyhemoglobin saturation (SpO_2_), and shock index were monitored for 60 min, and lung tissues were analyzed histopathologically. The study evaluated the effects of catecholamines as follows. Randomly assigned mice received 1.4 J/cm^2^ LISW followed by the immediate intraperitoneal administration of dobutamine, noradrenalin, or normal saline. The primary outcome was the survival rate. Additionally, sBP, HR, SpO_2_, and the shock index were measured before and 5 and 10 min after LISW, and the cardiac output, left ventricular ejection fraction, and systemic vascular resistance (SVR) were determined before and 1 min after LISW.

**Results:**

The triad of BLI (hypotension, bradycardia, and hypoxemia) was evident immediately after LISW. The survival rates worsened with increasing doses of LISW (100 % in 1.2 J/cm^2^ vs. 60 % in 1.3 J/cm^2^, 10 % in 1.4 J/cm^2^). The histopathological findings were compatible with those of human BLI. The survival rate in LISW high group (1.4 J/cm^2^) was highest in the group that received noradrenalin (100 %), with significantly elevated SVR values (from 565 to 1451 dyn s/min^5^). In contrast, the survival rates in the dobutamine and normal saline groups were 40 and 10 %, respectively, and the SVR values did not change significantly after LISW in either group.

**Conclusions:**

The main cause of death during the initial phase of severe BLI is hypotension due to the absence of peripheral vasoconstriction. Therefore, the immediate administration of noradrenalin may be an effective treatment during the initial phase of severe BLI.

## Background

Blast waves caused by explosions can cause death, even in the absence of external injuries [1, 2]. Physicians should be aware that blast injuries are not only an important cause of trauma in military conflicts, but also during acts of terrorism in civil settings. The physical damage inflicted by blast waves is called primary blast injury, which has become more prevalent because of recent changes in the characteristics of warfare and terrorism [3–6].

When blast waves pass from a solid into a gas-filled tissue interface, compressive stress is converted into a tension wave. Therefore, gas-filled organs, such as the lungs, gastrointestinal tract, and auditory system, are vulnerable to blast waves [2, 3, 6, 7], among which, blast injuries to the lungs can be the most lethal during the initial phase [7–12]. The severity of blast lung injuries (BLI) usually determines subsequent mortality [2].

Primary blast injury to the thorax produces the triad of BLI, namely, bradycardia [4, 11, 13], prolonged hypotension [4, 11, 13], and hypoxemia [8, 14]. Hypotension and hypoxemia due to BLI are the most serious life-threatening complications of primary blast injuries in initial survivors [5, 13]. However, rigorous efforts directed toward elucidating the etiology of hypotension have not yielded much information. Hence, treatment for severe BLI during the initial phase remains insufficient. There are currently arguments that hypotension might be mediated by the absence of vasoconstriction [13, 15, 16] and/or decreased cardiac output [16, 17]. Irwin et al. reported that blast-induced circulatory shock results from immediate myocardial depression without compensatory vasoconstriction [16]. However, none of these studies identified the effects of catecholamines, which are believed to increase vascular resistance and/or cardiac output.

Conventional models of blast injuries require the use of real explosives and complex shock tubes or blast generators to create shock waves. Such methods are rather costly and associated with physical hazards, which might have prevented the advancement of effective treatments for BLI. However, Satoh et al. recently introduced a novel laboratory-based small-animal model using laser-induced shock wave (LISW) [18, 19]. The LISW method is highly controllable and reproducible and can be implemented in simple experimental settings.

We hypothesized that the main cause of immediate death in individuals with severe BLI is hypotension due to an absence of peripheral vasoconstriction and that the immediate administration of an α1-adrenergic receptor agonist directly after exposure to blast waves may improve the acute prognosis. We thus examined the effects of catecholamines during the initial phase of severe BLI elicited by a high-dose LISW.

## Methods

### Animals

Inbred male 8- to 10-week-old C57BL/6 mice were housed under specific pathogen-free conditions in an environmentally controlled clean room under a 12-h light/dark schedule. All experiments proceeded according to the institutional ethical guidelines for animal experiments of the National Defense Medical College, and the Committee for Animal Research at the National Defense Medical College (Tokorozawa, Saitama, Japan) approved the study (permission number: 11045). The animals were planned to be sacrificed via 5 % sevoflurane administration followed by cervical dislocation when they demonstrated pain caused by their behaviors (struggling or screaming).

### Shock wave generator

**Fig. 1:**
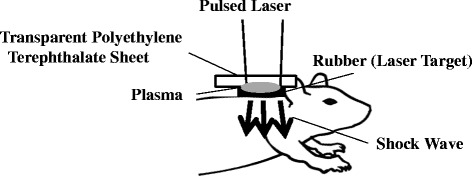
Experimental protocol for the mouse model of pulmonary blast injury using laser-induced shock wave (LISW) treatment. Black rubber laser target with a Q-switched ruby laser was irradiated to generate LISW. Plasma formed at the interface between the transparent material and black rubber. Patches were placed on the dorsal skin over the right and left lungs to generate bilateral lung damage

Briefly, LISW were generated by irradiating a laser target with a 694-nm Q-switched ruby laser (NIIC Co., Ltd., Tokyo, Japan) at a pulse width of 20 ns at full-width half-maximum (FWHM). The laser target was a 20-mm-diameter, 0.5-mm-thick black natural rubber disk, upon which a 1.0-mm-thick transparent polyethylene terephthalate sheet was bonded to confine the laser-induced plasma to increase the LISW impulse. The laser spot on the target was elliptical, and the longer and shorter axes were ~11 and ~9 mm, respectively (Fig. 1).

### Instrumentation and physiological measurements

The dorsal chest and neck regions of the mice were treated with a topical depilatory agent to avoid air becoming trapped in the fur and in preparation for the placement of the sensor over the carotid region. The ventral chest region was similarly prepared for echocardiography.

The mice continuously inhaled 1.5 % sevoflurane in air via a nose cone for anesthesia.

The peripheral oxyhemoglobin saturation (SpO_2_) and heart rate (HR) were measured using a MouseOX veterinary pulse oximeter (STARR Life Sciences Corp., Oakmont, PA, USA). Systolic blood pressure (sBP) was assessed using the MK-2000ST non-preheating, non-invasive blood pressure monitor system for mice and rats (Muromachi, Tokyo, Japan) while the mice were fixed on a plate in the prone position. The shock index was estimated using the equation: HR / sBP.

Cardiac output (CO) and the left ventricular ejection fraction (EF) were measured using an ellipsoid single-plane algorithm using a Vevo 770 high-resolution imaging system (Fujifilm VisualSonics Inc., Toronto, Canada) at 40 MHz with a frame rate of 30 Hz while the mice were fixed on a plate in the supine position. Systemic vascular resistance (SVR) was estimated using the equation: 80 × 1000 × (mean arterial pressure) / (stroke volume × HR) dyn s/cm^5^, where HR represents the heart rate.

### Experimental protocol

The mice were left to equilibrate for 15 min before baseline values for HR, sBP, SpO_2,_ shock index, EF, CO, and SVR were determined.

Most areas of the bilateral lungs were exposed to single LISW each (except for the Sham group). Ultrasound conductive gel placed between the laser target and skin surface ensured acoustic impedance matching.

### Validation of BLI induced by LISW

The mice were randomly allocated to receive 1.2 (LISW low group; *n* = 10), 1.3 (LISW medium group; *n* = 16), or 1.4 (LISW high group; *n* = 10) J/cm^2^ of LISW or no waves (Sham group; *n* = 7). The baseline sBP, HR, SpO_2_, and shock index values were measured before exposure to LISW. After delivering LISW twice at each dose, the survival rate, sBP, HR, SpO_2_, and shock index were measured every 5 min for 1 h. The mice were not intubated or ventilated in order to mimic clinically relevant chest trauma conditions.

Death was defined as the absence of an sBP measurement with no movements of any part of the body for >15 min.

### Histopathological characteristics

The mice were euthanized 1 h after LISW, and then isolated tracheas were cannulated with 22-gauge catheters. The lungs were inflated, fixed with 4 % paraformaldehyde at 10 cm H_2_O pressure, dehydrated, and sliced into sections for staining with hematoxylin-eosin. All experiments comprised at least four mice.

### Effects of noradrelalin and dobutamine on survival rates and physiological parameters

The baseline sBP, HR, SpO_2_, and shock index values were measured before LISW delivery, after which the mice were randomly allocated to the Sham group (*n* = 5), which received no LISW or drugs, a group that received 60 μg of dobutamine in 0.1 mL of saline (DOB group; *n* = 10), a group that received 20 μg of noradrenalin in 0.1 mL of saline (NA group; *n* = 10), or a group that received 0.1 mL of normal saline alone (NS group; *n* = 10) via intraperitoneal bolus injection immediately after delivering 1.4 J/cm^2^ LISW twice.

The sBP, HR, SpO_2_, and shock index were measured at 5 and 10 min after LISW in the dobutamine (DOB), noradrenalin (NA), and normal saline (NS) groups. The survival rates were calculated every 5 min for 1 h after LISW.

### Effects on hemodynamic parameters

The baseline sBP and HR values were measured before LISW. CO, EF, and SVR were calculated from the left ventricular end-diastolic and end-systolic volumes obtained using the ellipsoid single-plane algorithm. The mice received 1.4 J/cm^2^ LISW twice as described above and were then randomly allocated to the following groups: 60 μg of dobutamine in 0.1 mL of saline (DOB group; *n* = 5), 20 μg of noradrenalin in 0.1 mL of saline (NA group; *n* = 5), or 0.1 mL of normal saline alone (NS group; *n* = 5) via intraperitoneal bolus injection immediately after LISW.

### Statistical analysis

The baseline values for sBP, HR, shock index, and SpO_2_ obtained before LISW were assessed using a one-factor ANOVA. The baseline values of CO, EF, and SVR were assessed using the Kruskal-Wallis test. The survival periods were compared among the groups using the Kaplan-Meier method with the log-rank test. Changes in the sBP, HR, EF, and SVR values were assessed using a repeated measures ANOVA, paired *t* test, or Wilcoxon signed-rank test. The values are shown as the mean ± standard error. A *p* value of <0.05 was considered to be significant.

## Results

### Validation of BLI induced by LISW

As shown in Table 1, the baseline sBP, HR, SpO_2_, and shock index values did not differ significantly among the four groups.

**Table 1:** Baseline physiological data recorded before LISW

	Sham	1.2 J/cm^2^ (LISW low group)	1.3 J/cm^2^ (LISW medium group)	1.4 J/cm^2^ (LISW high group)	*p* value
*n*	7	10	16	10	
HR (beats/min)	530 ± 16.1	548 ± 13.5	531 ± 12.1	533 ± 14.7	0.801
sBP (mmHg)	83.9 ± 4.3	89.2 ± 4.5	84.2 ± 3.1	79.5 ± 4.1	0.428
SpO_2_ (%)	98.1 ± 0.6	98.3 ± 0.3	97.2 ± 0.5	97.2 ± 0.6	0.396
Shock index	6.06 ± 1.6	6.32 ± 0.4	6.42 ± 0.2	6.91 ± 0.5	0.451

*n* number of mice, *HR* heart rate, *sBP* systolic blood pressure, *SpO*_*2*_ peripheral oxyhemoglobin saturation, *n.s.* not significant (one-factor ANOVA). Data are presented as the mean ± standard error

**Fig. 2:**
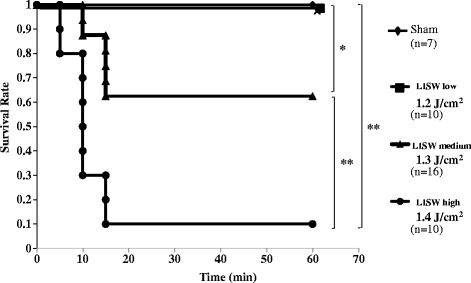
Kaplan-Meier survival curves for the mice exposed to 1.2, 1.3, and 1.4 J/cm^2^ of LISW. Data were recorded every 5 min for 60 min after the mice were exposed twice to 1.2 (LISW low group), 1.3 (LISW medium group), or 1.4 (LISW high group) J/cm^2^. Sham mice were not exposed to LISW. **p < 0.01; *p < 0.05 (log-rank test)

Figure 2 shows that the survival rates of each group at 1 h after LISW significantly decreased as the dose of LISW increased, and lethality was most evident within 15 min after LISW in mid-intensity (1.3 J/cm^2^) and high-intensity (1.4 J/cm^2^) groups, whereas none of the mice died after 15 min.

**Fig. 3:**
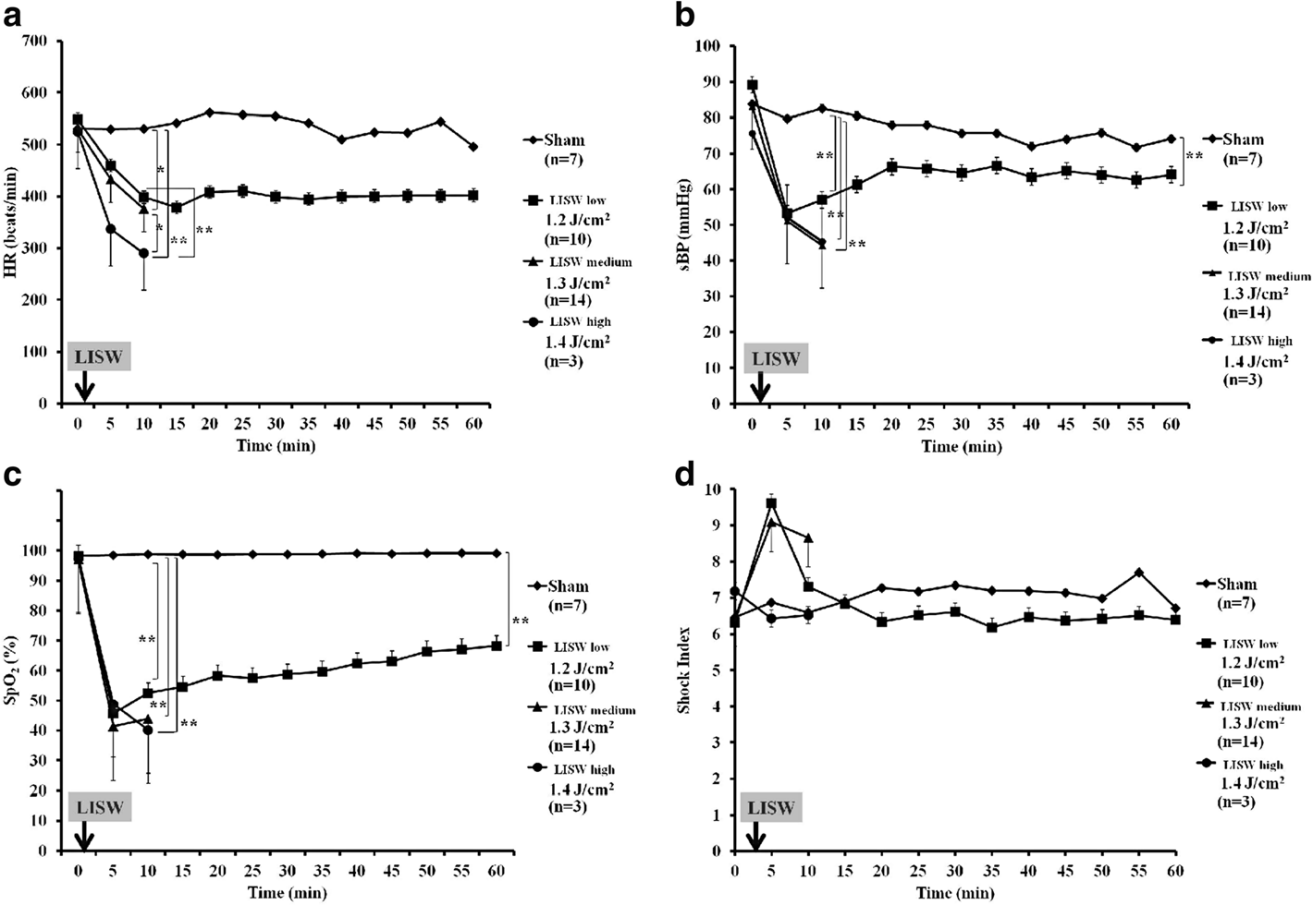
Effects of LISW on heart rate (HR), systolic blood pressure (sBP), peripheral oxyhemoglobin saturation (SpO_2_), and the shock index in the anesthetized mice. **a** HR data were recorded immediately before (0 min) and every 5 min up to 60 min after being exposed twice to LISW at 1.2 (LISW low group), 1.3 (LISW medium group; two mice that died within 10 min were excluded), and 1.4 (LISW high group; seven mice that died within 10 min were excluded) J/cm^2^. Sham mice were not exposed to LISW. *p* < 0.01 interaction by repeated measures ANOVA, **p < 0.01 for significance for LISW high group vs. LISW low group or Sham group, *p < 0.05 for significance for LISW medium group vs. LISW high group or Sham group determined according to Scheffe’s F test. The data are presented as the mean ± standard error. **b** sBP was recorded immediately before (0 min) and every 5 min up to 60 min being exposed twice to LISW at 1.2 (LISW low group), 1.3 (LISW medium group; two mice that died within 60 min were excluded), and 1.4 (LISW high group; seven mice that died within 10 min were excluded) J/cm^2^. Sham mice were not exposed to LISW. *p* < 0.01 interaction by repeated measures ANOVA, **p < 0.01 for significance for Sham group vs. LISW low group, medium group, or high group determined according to Scheffe’s F test. The data are shown as the mean ± standard error. **c** SpO_2_ in the anesthetized mice was assessed immediately before (0 min) and every 5 min up to 60 min after being exposed twice to LISW at 1.2 (LISW low group), 1.3 (LISW medium group; two mice that died within 60 min were excluded), and 1.4 (LISW high group; seven mice that died within 10 min were excluded) J/cm^2^. Sham mice were not exposed to LISW. *p* < 0.01 interaction by repeated measures ANOVA, **p < 0.01 for significance for Sham group vs. LISW low group, medium group, or high group determined according to Scheffe’s F test. The data are shown as the mean ± standard error. **d** The shock index in the anesthetized mice was assessed immediately before (0 min) and every 5 min up to 60 min after being exposed twice to LISW at 1.2 (LISW low group), 1.3 (LISW medium group; two mice that died within 60 min were excluded), and 1.4 (LISW high group; seven mice that died within 10 min were excluded) J/cm^2^. Sham mice were not exposed to LISW. There were no significant differences between the groups (repeated measures ANOVA). The data are shown as the mean ± standard error

As shown in Fig. 3a–c, sBP, HR, and SpO_2_ rapidly decreased soon after exposure to LISW. Low-intensity (1.2 J/cm^2^) LISW led transient shock at 5 min later LISW, when the shock index increased 1.5 times as that noted at baseline. Then, the sBP dropped to less than 60 mmHg and thereafter gradually increased again to more than 60 mmHg, while the shock index returned to the baseline level. In addition, LISW clearly decreased sBP, and it remained decrease for at least 1 h in LISW low group in Fig. 3a. SpO_2_ also remained decrease for at least 1 h in Fig. 3b.

Mid-intensity (1.3 J/cm^2^) LISW prolonged shock (sBP < 50 mmHg and shock index 1.5 times) beyond 10 min after LISW, and six of 16 mice died within 15 min.

High-intensity (1.4 J/cm^2^) LISW significantly attenuated the HR. Hence, the shock index decreased in a pseudonormal pattern and the sBP decreased to approximately 40 mmHg. Therefore, nine of the ten mice might have died because of sustained shock.

### Histopathological characteristics

**Fig. 4:**
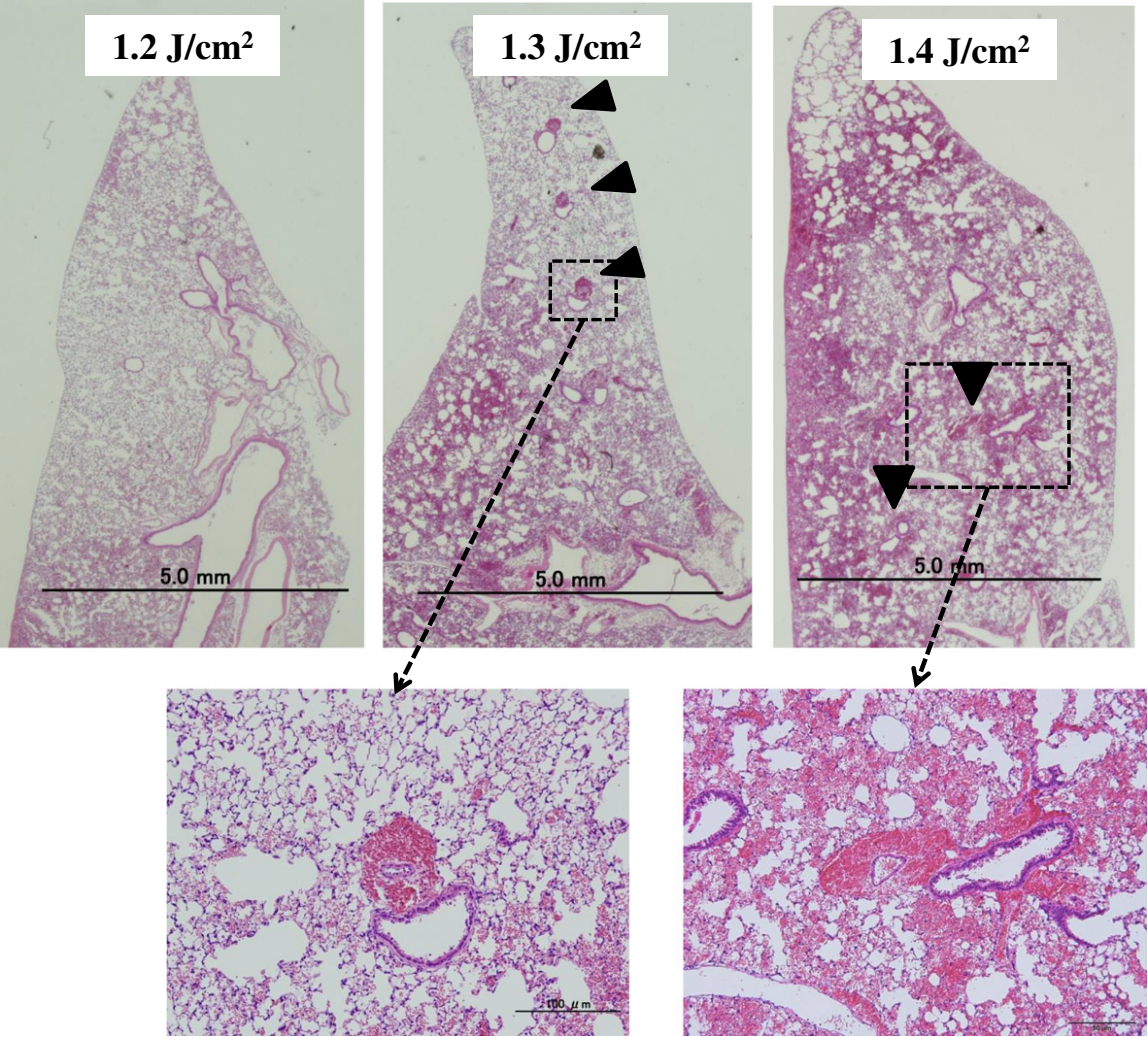
Representative sections of mouse lungs histologically stained with hematoxylin and eosin after exposure to 1.2, 1.3, and 1.4 J/cm^2^ of LISW. In each group, intra-alveolar and endobronchial hemorrhage and diffuse alveolar over-distension were evident. Perivascular cuff-like hemorrhage (*arrowheads*) were seen sporadically

Diffuse hemorrhage occurred in both lungs. An evaluation of lung samples using microscopy revealed endobronchial hemorrhage and diffuse alveolar over-distension (Fig. 4). In contrast, a few sites of perivascular (cuff-like) hemorrhage were evident (Fig. 4).

### Effects of noradrenalin and dobutamine on survival rates and physiological parameters

**Fig. 5:**
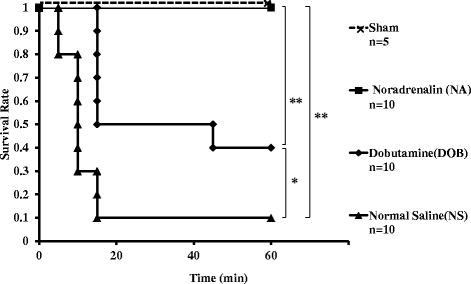
Kaplan-Meier survival curves for the mice exposed twice to 1.4 J/cm^2^ LISW and then administered a single intraperitoneal bolus of each drug. Data were recorded every 5 min for 60 min. Sham mice were not exposed to LISW and instead administered 0.1 mL normal saline. NA, DOB, and NS groups were exposed twice to LISW and then received 20 μg of noradrenaline, 60 μg of dobutamine, and 0.1 mL of normal saline, respectively. **p < 0.01; *p < 0.05 log-rank test

The baseline sBP, HR, SpO_2_, and shock index values were at the same levels in the dobutamine (DOB), noradrenalin (NA), and normal saline (NS) groups (Table 2). The survival rates in both catecholamine groups were significantly higher than that seen in the NS group after exposure to 1.4 J/cm^2^ of LISW (Fig. 5). All Sham animals survived until the end of the study. Nine (90 %) of the NS group mice died within 15 min after LISW. Four (0 %) mice in the DOB group survived until the end of the study. One (10 %) mouse died after 45 min, and five (50 %) mice died within 15 min. All mice in the NA group survived until the end of the observation. The survival rates were significantly higher in the NA and DOB groups than in the NS group (*p* < 0.01 and *p* < 0.05, respectively) and in the NA group than in the DOB group (*p* < 0.01).

**Table 2:** Baseline physiological data recorded before LISW

	Sham	Normal saline (NS group)	Noradrenalin (NA group)	Dobutamine (DOB group)	*p* value
*n*	5	10	10	10	
HR (beats/min)	530 ± 16.1	525.8 ± 20.1	515 ± 21.8	414 ± 29.8	0.941
sBP (mmHg)	83.9 ± 4.3	83.4 ± 3.4	79.8 ± 4.8	95 ± 4.6	0.069
SpO_2_ (%)	98.1 ± 0.6	98.3 ± 0.3	97.2 ± 0.5	97.2 ± 0.6	0.282
Shock index	6.06 ± 1.6	6.32 ± 0.4	6.54 ± 0.48	5.52 ± 0.24	0.193

*n* number of mice, *HR* heart rate, *sBP* systolic blood pressure, *SpO*_*2*_ peripheral oxyhemoglobin saturation, *n.s.* not significant (one-factor ANOVA). Data are presented as the mean ± standard error

**Fig. 6:**
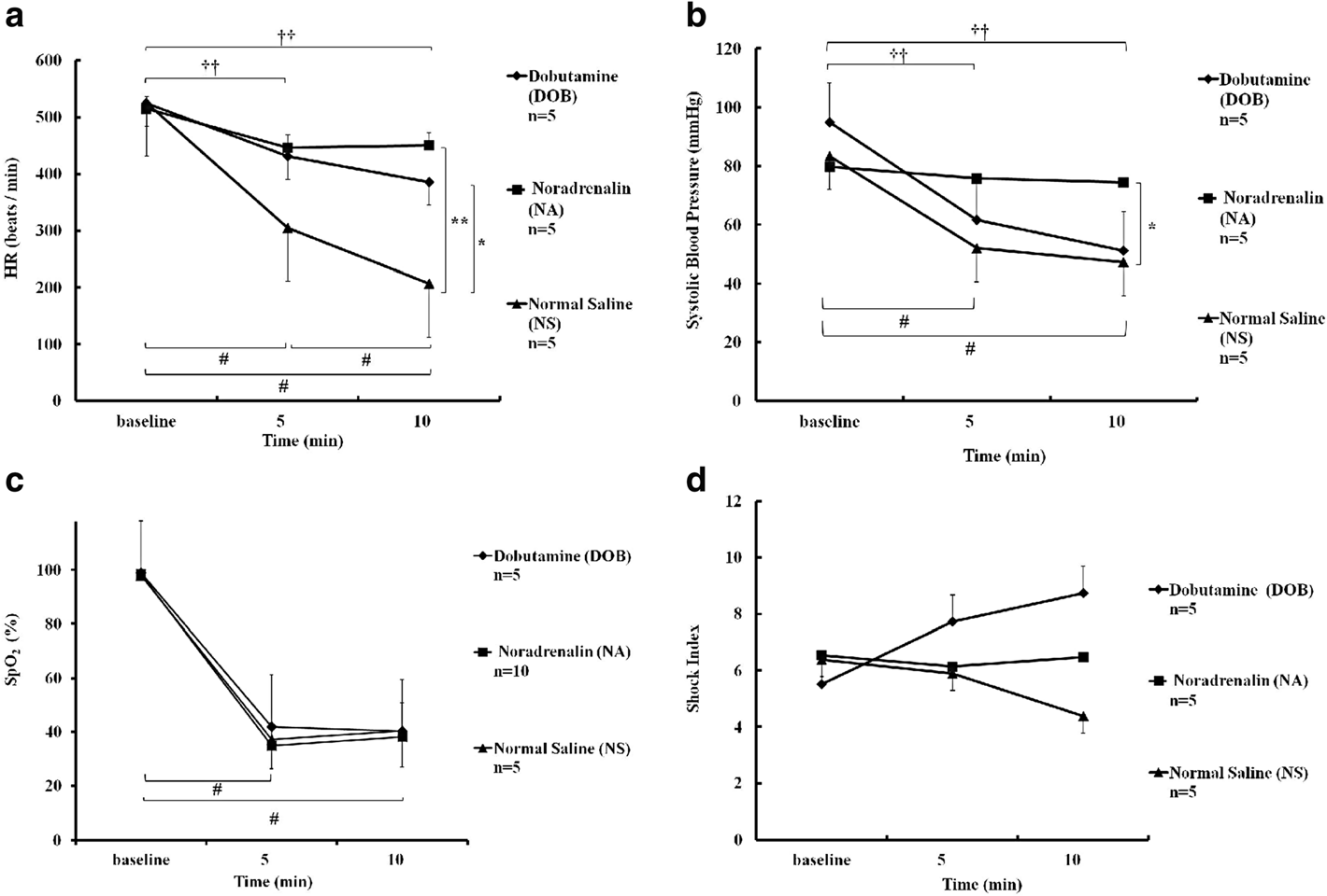
Effect of the drugs administered to the mice immediately after being exposed twice to LISW at 1.4 J/cm^2^ on systolic blood pressure (sBP), heart rate (HR), peripheral oxyhemoglobin saturation (SpO_2_), and the shock index. **a** HR was measured immediately before (baseline) and at 5 and 10 min after LISW. NA, DOB, and NS groups were exposed twice to LISW and then received 20 μg of noradrenaline, 60 μg of dobutamine, and 0.1 mL of normal saline, respectively. *p* < 0.01 interaction by repeated measures ANOVA, **p < 0.01; *p < 0.05 for significance between two groups determined according to Scheffe’s F test. #*p* < 0.05; significance between each value in NS group, baseline, and 5 min in NA group, and corresponding vs. 10 min-value in DOB group (Wilcoxon signed-rank test). ††*p* < 0.01; significance between baseline and 10 min in NA group, baseline, and 5 min in DOB group (paired *t* test). The data are shown as the mean ± standard error. **b** sBP was measured before (baseline) and at 5 and 10 min after LISW. NA, DOB, and NS groups were exposed twice to LISW and then received 20 μg of noradrenaline, 60 μg of dobutamine, and 0.1 mL of normal saline, respectively. *p* < 0.05 interaction by repeated measures ANOVA, *p < 0.05 for significance between two groups determined according to Scheffe’s F test. #*p* < 0.05; significance corresponding vs. baseline values in NS group (Wilcoxon signed-rank test). ††*p* < 0.01; significance corresponding vs. baseline value in DOB group (paired *t* test). **c** SpO_2_ was measured immediately before (baseline) and at 5 and 10 min after LISW. #*p* < 0.01 vs. corresponding baseline values in all groups (paired *t* test). **d** The shock index was measured immediately at baseline and 5 and 10 min after LISW. There were no significant differences between the groups or based on time

For the analysis of physiological parameters, the mice for which we could not obtain both sBP and HR values were excluded (five mice were excluded in each group) because we could not measure sBP, HR, or SpO_2_ in five mice of the NS or DOB group due to peripheral circulatory failure. Moreover, we failed to measure sBP and HR using a non-invasive blood pressure monitor system in five mice of the NA group. HR significantly decreased in all groups after 5 and 10 min of LISW, although NA and DOB mitigated bradycardia in comparison with that observed in the NS group (*p* < 0.01 and *p* < 0.05, respectively) (Fig. 6a). NA administration maintained the sBP values above 70 mmHg (Fig. 6b) and resulted in the same shock index (Fig. 6d), even after LISW, while the sBP values were not more than 55 mmHg in the other groups (Fig. 6b). As a result, DOB did not maintain the hemodynamics, and the shock index increased 1.5 times the baseline level (Fig. 6d). As for the NS group, severe bradycardia occurred, as in validation study (Fig. 6a), and the shock index decreased in a pseudonormal pattern (Fig. 6d).

The SpO_2_ at 5 min similarly and significantly decreased after LISW in all three groups and did not change for up to 10 min thereafter (Fig. 6c).

### Effect on hemodynamic parameters

**Fig. 7:**
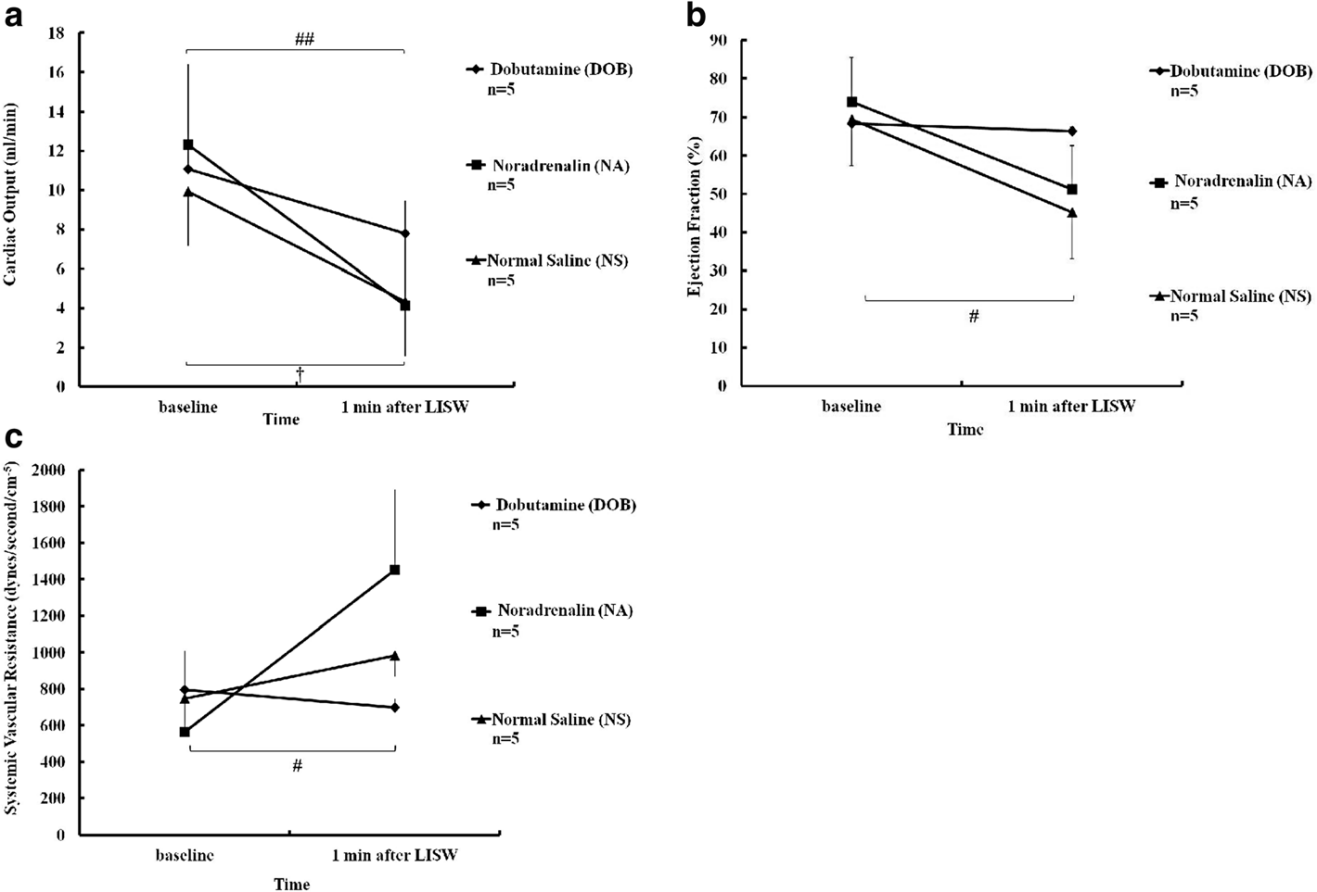
Effect of the drugs administered to the mice immediately after two exposures to 1.4 J/cm^2^ LISWs on cardiac output (CO), left ventricular ejection fraction (EF), and systemic vascular resistance (SVR). **a** CO was determined before (baseline) and at 1 min after LISW. NA, DOB, and NS groups were exposed twice to LISW and then received 20 μg of noradrenaline, 60 μg of dobutamine, and 0.1 mL of normal saline, respectively. ##*p* < 0.01 vs. corresponding baseline values in NA (Wilcoxon signed-rank test). †*p* < 0.05 vs. corresponding baseline values in NS groups (paired *t* test). The data are shown as the mean ± standard error. **b** EF was determined before (baseline) and at 1 min after LISW. NA, DOB, and NS groups were exposed twice to LISW and then received 20 μg of noradrenaline, 60 μg of dobutamine, and 0.1 mL of normal saline, respectively. #*p* < 0.05 vs. corresponding baseline values in NA or NS groups (Wilcoxon signed-rank test). The data are shown as the mean ± standard error. **c** SVR was determined before (baseline) and at 1 min after LISW. NA, DOB, and NS groups were exposed twice to LISW and then received 20 μg of noradrenaline, 60 μg of dobutamine, and 0.1 mL of normal saline, respectively. #*p* < 0.05 vs. baseline value in NA group (Wilcoxon signed-rank test). The data are shown as the mean ± standard error

The baseline EF, CO, and SVR values did not differ significantly among the groups (Table 3). EF and CO modestly decreased in the DOB group after LISW (68 to 66 %, 11.1 to 7.8 ml/min), whereas both significantly decreased in the NA and NS groups (74 to 51 %, 12.3 to 4.1 ml/min, 69 to 45 %, 9.9 to 4.3 ml/min) (Fig. 7a, b). DOB preserved CO and EF, as DOB stimulated cardiac contractility. NA administration significantly increased SVR 2.6-fold the baseline level, even after LISW exposure (565 to 1451 dyn s/min^5^, *p* < 0.05) (Fig. 7c), whereas the SVR values remained unchanged in the DOB and NS groups (796 to 699, 747 to 983 dyn s/min^5^, respectively) (Fig. 7c).

**Table 3:** Baseline cardiac data recorded before LISW

	Normal saline (NS group)	Noradrenalin (NA group)	Dobutamine (DOB group)	*p* value
*n*	5	5	5	
CO	9.9 ± 1.2	12.3 ± 1.0	11.1 ± 2.9	0.688
EF	69.4 ± 2.3	74 ± 2.2	68.4 ± 2.2	0.276
SVR	747 ± 72	565 ± 30	796 ± 158	0.166

*n* number of mice, *CO* cardiac output, *EF* left ventricular ejection fraction, *SVR* systemic vascular resistance, *n.s.*, not significant (Kruskal-Wallis test). Data are presented as the mean ± standard error

## Discussion

Although the rate of primary blast injury (damage caused by blast waves) has historically been lower than that of secondary blast injury (damage caused by projectiles propelled by blast wind), this rate is currently approaching that of secondary blast injury. Studies published before the twenty-first century show rates of primary blast injury between 0.9 and 8.4 % [20, 21], whereas recent reports indicate rates between 14 and 86 % [1, 2, 4, 20]. One explanation for why the clinical importance of BLI in warfare has been increasing is the increased prevalence of personal body armor for soldiers capable of protecting against secondary, but not primary, blast injuries [6, 7, 22]. In civilian settings, terrorists tend to set bombs in confined spaces, such as trains, busses or buildings in order to magnify the duration and range of blast waves by forcing energy to rebound off surfaces [1, 5–7, 9, 20, 23].

Most deaths due to BLI occur immediately after blast exposure during the initial phase (prehospital setting) [7–10, 20, 24]. Although no documents have clearly defined the duration immediately after injury, it is generally considered that 15–30 min is necessary to evacuate the patient to the hospital. In the present study, we defined the initial phase as within 30 min after injury, which is in agreement with the fact that most of the mice died within 15 min after LISW in this study. Our model therefore seems to be compatible with the characteristics of severe BLI. In addition, BLI is clinically characterized by the triad of bradycardia, hypotension, and hypoxemia. We also found this triad in validation study.

The micromorphological equivalents of BLI can be summarized as including diffuse alveolar over-distension, alveolar and endobronchial hemorrhage, circumscribed interstitial hemorrhage with a cuff-like pattern around pulmonary vessels, venous air embolism, and pulmonary fat embolism [25]. We found diffuse alveolar over-distension and intra-alveolar, endobronchial, and perivascular (cuff-like) hemorrhage in this model.

The etiology of the hypotension seen after BLI is complex. Previous studies suggest that it is due to the absence of peripheral vasoconstriction [13, 15, 16] caused by inhibition of the sympathetic vasoconstrictor tone [26, 27] or release of potent vasodilator nitric oxide [28, 29]. However, few studies have documented the details of the hemodynamic response, especially regarding cardiac contractions and SVR, to catecholamine administration after blast lung injury.

Indeed, we found that the SVR values did not change before and after LISW exposure despite severe hypotension in the NS and DOB groups. We evaluated the effects of noradrenaline, an α1-adrenergic receptor agonist that increases vascular resistance and a β1-adrenergic receptor agonist that preserves CO, and dobutamine, a β1-adrenergic receptor agonist, during the initial phase of severe BLI. No compensatory increases in SVR occurred after LISW exposure in the NS group, and very low cardiac output caused hypotension. Although DOB preserved CO and EF, it did not prevent hypotension, as DOB stimulated cardiac contractility via the β1-adrenergic receptor, although it did not affect peripheral vasoconstriction, as shown by the unchanged SVR values. In contrast, NA increased SVR, stimulating peripheral vasoconstriction via the α1-adrenergic receptor, which consequently maintained sBP. Therefore, hypotension due to a lack of compensating vasoconstriction is the essential pathophysiology and therapeutic target of BLI. In addition, NA is also a β1-adrenergic receptor agonist, but we think that the α1-adrenergic effect is a major factor affecting survival and BP maintenance immediately after LISW exposure because dobutamine, a β1-adrenergic receptor agonist, could not maintain sBP in our model.

The survival rate was significantly higher in the noradrenaline group than in the dobutamine group, indicating that hypotension is the main cause of death due to BLI during the initial phase. The lack of peripheral vasoconstriction, rather than decreased CO, is the main cause of the hypotension associated with BLI. Compensatory vasoconstriction adjusts the blood distribution from peripheral vessels toward central vital organs, such as the brain and heart. NA administration rationally allows for the blood to be distributed throughout the central vital organs, subsequently preventing low cardiac output associated with severe bradycardia. However, DOB administration simultaneously increases O_2_ demand because of the positive inotropic effect, which fails to improve the acute survival rates. Therefore, NA, not DOB, acts as an effective treatment for the initial phase of severe BLI.

In addition, LISW clearly decreases sBP, and it remains decreased for at least 1 h in the LISW low group. We do not know the mechanism, but lung damage based on LISW exposure may influence the cardiac function because SpO_2_ also remains decreased for at least 1 h. Right heart insufficiency might occur and the reduction of venous return may have influenced the decreased sBP in mice.

Clinically, no treatment has been established for BLI during the initial phase, such as that observed in the prehospital or battlefield setting. If the immediate administration of catecholamines is effective for victims of severe BLI during the initial phase, then the intra-muscular injection of adrenaline might be a practical treatment, as it is already easily, safely, and effectively administered to anaphylactic patients in prehospital settings [30]. The present findings suggest that the administration of α1-adrenergic receptor agonists may be an effective and rational initial treatment for severe BLI.

### Limitations

We applied LISW twice to mice in order to create bilateral BLI, whereas most victims of explosives are exposed to only one blast wave. This experimental model might therefore differ from real situations to some extent. However, we believe that the difference is permissible because all mice exposed to LISW developed the same BLI triad of hypotension, bradycardia, and hypoxemia.

Although bradycardia may have affected the survival rate to some extent, we did not evaluate the effect of atropine because atropine administration did not result in any significant increases in HR in the C57BL/6 Sham group. Therefore, we evaluated only the effects of noradrenaline and dobutamine.

In addition, another major limitation of this study was the timing of intervention. It is unlikely that an individual with BLI will receive noradrenalin within several minutes after injury. If noradrenalin was not administered immediately after LISW, nine of ten mice died within 15 min after LISW exposure in our model. Thus, a rapid injection of noradrenalin is necessary for lifesaving according to our experiments. We will conduct further studies to develop a rapid injection system of noradrenalin for use after an actual explosion.

## Conclusions

The main cause of immediate death from BLI may be hypotension due to the absence of peripheral vasoconstriction. Therefore, the immediate administration of α1-adrenergic receptor agonists, such as noradrenaline, might serve as an effective initial treatment for severe BLI.

## Abbreviations

BLI: blast lung injury
CO: cardiac output
DOB: dobutamine
EF: ejection fraction
HR: heart rate
LISW: laser-induced shock wave
NA: noradrenalin
NS: normal saline
sBP: systolic blood pressure
SpO_2_: peripheral oxyhemoglobin saturation
SVR: systemic vascular resistance
